# Analysis of Al_2_O_3_ Single-Bead Deposition Behavior and Microstructure on a Ti-6Al-4V Substrate Using the Laser-Directed Energy Deposition (DED-LB) Process

**DOI:** 10.3390/ma19112369

**Published:** 2026-06-02

**Authors:** Tae-Hyeon Kim, Jin-Soo Lee, Sang-In Kim, Su-Han Bae, Changjong Kim, Se-Yun Kim

**Affiliations:** 1 Department of Mechatronics Engineering, Kyungnam University, Changwon 51767, Republic of Korea; kth039976@gmail.com (T.-H.K.); su258369@naver.com (J.-S.L.); ksi980608@naver.com (S.-I.K.); suhan0125@naver.com (S.-H.B.); 2 Digital Manufacturing Innovation Division, Research Institute of Medium & Small Shipbuilding, Busan 46757, Republic of Korea; 3 Department of Future Automotive Engineering, Kyungnam University, Changwon 51767, Republic of Korea

**Keywords:** laser-directed energy deposition (DED-LB), Ti-6Al-4V (Ti64), Al_2_O_3_, additive manufacturing, dilution ratio

## Abstract

Al_2_O_3_ single beads were deposited on a Ti-6Al-4V (Ti64) substrate by laser-directed energy deposition (DED-LB) to establish baseline process conditions for ceramic protective layers and future Ti64/Al_2_O_3_ functionally graded materials (FGMs). These ceramic-containing surface layers are applicable to titanium components requiring improved oxidation, wear, and thermal resistance in aerospace, automotive, and high-temperature structural applications. Laser power (300–700 W) and scan speed (300–700 mm/min) were varied, and bead geometry was quantified from cross-sectional observations; energy density and dilution ratio were calculated. Melt pool depth increased with higher power and lower speed, indicating increased heat input and substrate melting. Crack formation in the melt zone was more sensitive to laser power than to scan speed. In contrast, bead height showed a non-monotonic response to energy density, which may be associated with possible coupled effects such as recoil pressure-driven melt pool disturbance, powder scattering, and insufficient powder melting at high scan speeds. Dilution-based optimization identified 300 W laser power and 400 mm/min scan speed, with a powder feed rate of 3 g/min, as the most suitable condition within the investigated process window, giving the lowest practical dilution ratio of approximately 40.27%. SEM–EDS and XRD analyses were conducted to examine the interfacial microstructure and phase characteristics under the selected condition. Overall, this study provides fundamental process guidelines and mechanistic insight into bead formation, dilution behavior, and interface formation, supporting the future application of DED-LB-based ceramic protective or graded layers on Ti64 surfaces.

## 1. Introduction

Additive manufacturing is a process used to fabricate three-dimensional structures by depositing materials layer by layer according to a designed geometry [1]. Among metal additive manufacturing technologies, laser-directed energy deposition (DED-LB) is a process in which powder feedstock is supplied into a melt pool generated by a focused high-power laser beam on a substrate. Owing to its flexible material feeding, relatively high deposition rate, and applicability to curved or localized surfaces, DED-LB is widely used for large-scale deposition, repair, remanufacturing, and surface modification of engineering components [2,3]. Ahn reported that DED-LB is advantageous for repair and multi-material deposition because energy input and material supply can be controlled simultaneously; however, the process still requires careful control of melt-pool stability, residual stress, dimensional accuracy, and defect formation [3]. Piscopo and Iuliano emphasized the industrial potential of laser powder-based DED-LB for repair and surface functionalization, although most reported applications have focused on metallic systems rather than metal–ceramic deposition [4]. Aprilia et al. also demonstrated the usefulness of DED-LB for restoring damaged engineering components, but the interfacial instability and cracking issues associated with ceramic-containing deposited layers were not fully addressed [5]. These studies indicate that DED-LB is a promising process for repair and surface functionalization, but they also reveal the need for further investigation of dissimilar metal–ceramic systems.

Recent studies have further highlighted the importance of process stabilization, melt-pool control, and defect reduction in laser-based manufacturing. Mohsan et al. showed that resonant ultrasonic vibration-assisted laser cladding can modify molten-pool behavior and improve the microstructure and performance of laser-cladded coatings [6]. In addition, recent DED repair studies using defocus control and thermodynamic modeling have demonstrated that process-parameter optimization is critical for stable track formation and repair geometry control [7]. However, these studies mainly focused on metallic coatings, repair applications, or auxiliary process control, whereas the fundamental deposition behavior of ceramic powder on Ti64 substrates remains insufficiently understood.

Ti alloys are widely used as structural materials in the automotive, aerospace, and biomedical industries because of their low density, high specific strength, corrosion resistance, and biocompatibility [8]. Among them, Ti-6Al-4V (Ti64) is one of the most representative α + β titanium alloys and is widely applied to components requiring a high strength-to-weight ratio and reliable corrosion resistance [9,10,11]. However, the long-term use of Ti64 in high-temperature environments is limited by surface degradation caused by oxidation [12]. When Ti64 is exposed to high-temperature oxidizing conditions, a TiO_2_ layer forms on the surface. Although this oxide layer can initially act as a protective scale, its continued growth may generate internal stress because of the thermal expansion mismatch between Ti64 and TiO_2_. This stress can cause cracking and spallation of the oxide layer, and repeated oxidation–spallation cycles can accelerate surface damage and reduce component lifetime [12]. Therefore, surface protection strategies that can improve the oxidation, thermal, and wear resistance of Ti64 are required to expand its applicability to high-temperature components [13].

Al_2_O_3_ is a representative ceramic material for such protective applications because of its high thermal stability, oxidation resistance, corrosion resistance, and wear resistance [14,15,16]. When applied as a surface layer, Al_2_O_3_ can suppress oxidation and thermal damage of metallic substrates in harsh environments. In this regard, DED-LB offers a potential route for forming ceramic-containing protective layers or compositionally graded structures on Ti64 surfaces by controlling the supplied powder and laser energy input [17,18,19,20]. Compared with conventional coating or complete component replacement approaches, DED can locally deposit functional materials only where needed, which may reduce processing time, material consumption, and maintenance cost while extending the service life of high-value components.

However, DED of metal–ceramic systems is challenging because of the large differences in thermophysical properties between metals and ceramics. In the Ti64/Al_2_O_3_ system, differences in melting point, thermal conductivity, coefficient of thermal expansion, and wettability can cause unstable melt-pool behavior, insufficient powder melting, excessive dilution, crack formation, and weak interfacial bonding [21,22,23,24,25]. In addition, the inherent brittleness of ceramic materials and the rapid heating–cooling cycle of the laser process can increase residual stress during deposition and promote crack formation. Therefore, before fabricating multilayer ceramic coatings or Ti64/Al_2_O_3_ functionally graded materials, it is necessary to clarify the single-bead track formation behavior and establish an effective process window [26,27,28].

In this study, Al_2_O_3_ single beads were deposited on a Ti64 substrate using the DED-LB process by varying laser power and scan speed. The melt pool depth, bead height, energy density, and dilution ratio were measured from cross-sectional observations to evaluate the effects of process parameters on bead formation and bonding behavior. In addition, SEM–EDS and XRD analyses were conducted under the selected condition to examine the interfacial microstructure and possible phase characteristics. The objective of this study is not merely to fabricate Al_2_O_3_ beads, but to establish baseline process conditions and mechanistic understanding for future DED-LB-based ceramic protective layers and Ti64/Al_2_O_3_ functionally graded materials.

## 2. Materials and Methods

The base material used in this experiment was a Ti-6Al-4V (Ti64) substrate, commonly referred to as Grade 5 titanium alloy, with dimensions of 150 × 50 × 3 mm. Ti64 is an α + β titanium alloy with a chemical composition of 5.5–6.75 wt.% Al, 3.5–4.5 wt.% V, minor amounts of Fe, O, C, N, and H, and balance Ti. Its typical properties include a density of approximately 4.43 g/cm^3^, an elastic modulus of about 110–114 GPa, a melting range of approximately 1600–1660 °C, a thermal conductivity of approximately 6.7–7.3 W/m·K, and a coefficient of thermal expansion of approximately 8.6–9.1 × 10^−6^ K^−1^. These physical, mechanical, and thermal properties are relevant to the present Ti64/Al_2_O_3_ deposition system because the thermophysical mismatch between Ti64 and Al_2_O_3_ can strongly influence melt-pool behavior, dilution, residual stress, and crack formation during DED-LB processing. In the DED-LB process, powder flowability is directly related to stable supply to the powder feeder, and generally, spherical powder has better flowability than irregularly shaped powder, resulting in higher supply stability [28]. Additionally, if powder particles are too large, complete melting is difficult, which can lead to melting failures; conversely, if particles are too small, excessive heat absorption can increase spatter during the process [29,30]. Therefore, in this study, to improve powder supply stability during the DED-LB process, spherical Al_2_O_3_ powder (Denka Co., Ltd., Tokyo, Japan) was sieved prior to deposition to use a particle size fraction of 45–150 μm. The morphology of the powder was observed using a scanning electron microscope (SEM; S-4200, Hitachi, Tokyo, Japan) and confirmed to be generally spherical.

In order to analyze the DED-LB deposition tendency of Al_2_O_3_ powder on a Ti64 substrate, a single-bead experiment was conducted to form a single track on the base material and evaluate the effects of process conditions on bead shape and deposition quality [31]. A DED-LB system (MX-450, InssTek, Daejeon, Republic of Korea) was used for the experiment. The MX-450 is a 3 + 2-axis DED-LB system with a 1 kW Ytterbium fiber laser (Gaussian profile), a wavelength of 1070 nm, a working area of 450 × 450 × 350 mm, and a laser beam diameter of 0.8 mm. The main process variables include laser power, scan speed, and powder feed amount, and among them, the effects of laser power and scan speed on the Al_2_O_3_ deposition tendency and the formation of the bonding interface were analyzed.

In this experiment, the fixed process conditions were set as follows: powder feed rate 3 g/min, powder gas 2.5 L/min, coaxial gas 7.0 L/min, shield gas 5.0 L/min, and laser beam diameter 0.8 mm. Laser power and scan speed were selected as process variables, and deposition was performed while varying the laser power in the range of 300–700 W at 100 W intervals and the scan speed in the range of 300–700 mm/min at 100 mm/min intervals.

To evaluate the cross-sectional shape and bonding behavior of the deposited beads under each process condition, the specimens were cut and polished. The cutting was performed using a cutting device (Secotom-15, Struers, Copenhagen, Denmark) and a diamond wheel (127 mm dia. × 0.4 mm × 12.7 mm, M0D13, Struers, Copenhagen, Denmark). After cutting, the cross-section was observed with an optical microscope (AX70TRF, Olympus, Tokyo, Japan). Bead geometry was measured from the optical micrographs using Image-Pro software (version 11.0.2, Media Cybernetics, Rockville, MD, USA). Bead height was defined as the vertical distance from the original substrate surface to the top of the deposited bead, whereas melt pool depth was defined as the maximum vertical penetration depth of the melted substrate region below the original substrate surface. Bead width was defined as the lateral width of the deposited bead measured at the substrate surface level. These geometric parameters were used to compare the effects of laser power and scan speed on bead formation, substrate melting, and dilution. SEM and energy dispersive X-ray spectroscopy (EDS; S-3500N, Hitachi, Tokyo, Japan) were utilized to evaluate the microstructure and element distribution formed at the bonding interface of the deposited beads. In addition, because reaction phases between Ti64 and Al_2_O_3_ or Ti oxides can affect the quality and reliability of the deposited beads by changing interfacial bonding strength and crack propagation paths, the possible presence of these reaction phases and oxides was examined using X-ray diffraction (XRD; MiniFlex, Rigaku, Tokyo, Japan) analysis [32,33].

## 3. Results and Discussion

**Figure 1:**
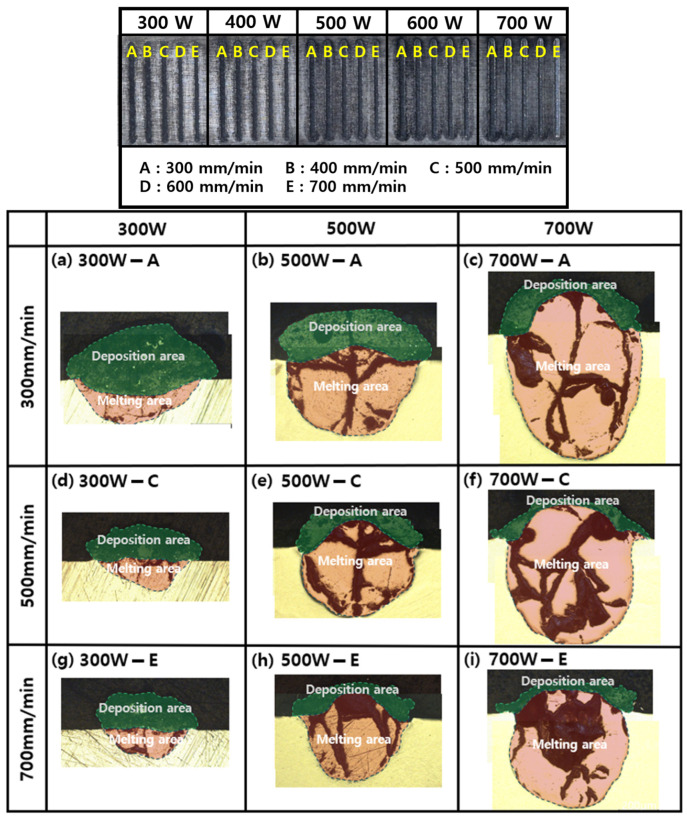
Photograph of Al_2_O_3_ Single Bead viewed from above and cross-section of Al_2_O_3_ Single Bead observed using an optical microscope (**a**) 300 W—300 mm/min, (**b**) 500 W—300 mm/min, (**c**) 700 W—300 mm/min, (**d**) 300 W—500 mm/min, (**e**) 500 W—500 mm/min, (**f**) 700 W—500 mm/min, (**g**) 300 W—700 mm/min, (**h**) 500 W—700 mm/min, (**i**) 700 W—700 mm/min.

Figure 1 shows top-view images and cross-sectional optical micrographs of the Al_2_O_3_ single beads deposited under different laser power and scan speed conditions. Under all processing conditions, cracks and irregular surface morphologies were observed in the deposited beads and melted regions. In several cross-sections, locally missing regions were also observed; these regions are interpreted as material detachment that occurred during cutting and polishing along pre-existing cracks generated during the deposition process.

**Figure 2:**
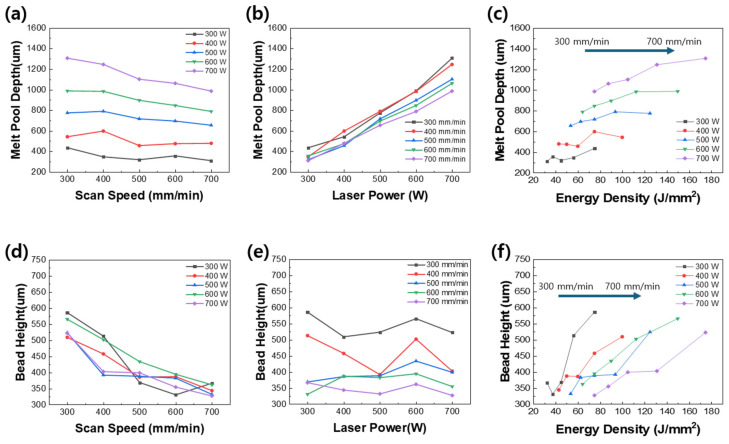
Melt pool depth depending on (**a**) scan speed, (**b**) laser power, and (**c**) energy density and bead height depending on (**d**) scan speed, (**e**) laser power, and (**f**) energy density.

Based on the cross-sectional optical micrographs, bead height, bead width, and melt pool depth were measured for each processing condition, and the areal energy density and dilution ratio were calculated. The measured and calculated results are summarized in Table 1. To further examine the deposition behavior of Al_2_O_3_ single beads, the variations in melt pool depth and bead height with laser power, scan speed, and areal energy density are plotted in Figure 2.

As shown in Figure 1a,e,i, different cross-sectional morphologies were obtained even under the same energy density condition. This indicates that energy density alone cannot fully describe the bead formation behavior in the present Ti64/Al_2_O_3_ deposition system. The quantitative results in Table 1 further show that the average melt pool depth increased markedly as the laser power increased, whereas the effect of scan speed was relatively smaller within the investigated process window. Specifically, the average melt pool depth increased from approximately 353 μm to 1140 μm as the laser power increased from 300 W to 700 W. These results indicate that melt pool formation and substrate melting were more strongly governed by laser power than by scan speed under the present experimental conditions.

Figure 2a shows that the molten pool depth increases with increasing laser power and decreasing laser scan speed. As laser power increases or laser scan speed decreases, the laser energy per unit area received by the substrate increases. In other words, increasing laser power or decreasing laser scan speed increases energy density.

Energy density is the laser energy supplied per unit area and increases with higher laser power, lower scan speed, and smaller laser beam size. In this study, the energy density was calculated as a process parameter using the laser power, scan speed and laser beam size, as shown in Equation (1). Differences in laser absorptivity between Ti64 and Al_2_O_3_ were not included in this calculation; therefore, the calculated value should be interpreted as the input energy density rather than the actual absorbed energy density of each material.

(1) $$E_{f}=\frac{P}{V\times f}$$

In the energy density calculation formula, *P*, *V*, and *f* are defined as the laser power, scan speed, and laser beam diameter, respectively [34]. As the energy density increases, the heat input to the substrate increases, thereby promoting substrate melting and increasing the melt pool depth. In addition, Marangoni convection induced by surface-tension gradients within the melt pool may become stronger under high-energy-input conditions, promoting downward melt flow and deeper melt pool formation.

When the energy density exceeds a certain level, localized evaporation of the Ti64 substrate can occur, generating a high-pressure vapor plume. The reaction force exerted by the vaporized material on the melt pool surface, referred to as recoil pressure, can depress the melt pool surface, increase melt pool penetration, and induce keyhole-like instability [35,36]. The expansion of the vapor plume may also disturb the supplied powder stream, causing Al_2_O_3_ particles to be deflected or scattered away from the melt pool. These phenomena can reduce powder capture efficiency and contribute to irregular bead morphology, pore formation, and cracking under high-energy-density conditions [37]. As shown in Figure 1, high-energy-density conditions generally produced deeper and less stable melt pools. However, because direct in situ melt pool observation was not performed in this study, keyhole-like instability and recoil-pressure-induced melt pool fluctuation should be interpreted as plausible mechanisms rather than directly confirmed phenomena.

In contrast to melt pool depth, bead height did not show a clear monotonic trend with laser power, scan speed, or energy density, as shown in Figure 2b. Figure 1 also shows that the Al_2_O_3_ deposition area tended to decrease with increasing laser power. This behavior may be associated with the low wettability between Al_2_O_3_ and the Ti64 melt, as well as recoil-pressure-induced melt pool disturbance, which can cause Al_2_O_3_ powder to be repelled from the melt pool or swept toward the bead edge, thereby reducing effective deposition [38]. Moreover, higher laser power increases the degree of Ti64 substrate melting, which increases the volume fraction of the Ti64-rich melt under the same powder feed condition and consequently reduces the apparent Al_2_O_3_ deposition area.

A reduction in the Al_2_O_3_ deposition area was also observed at higher scan speeds. Faster scan speeds reduce the heat input per unit length and shorten the lifetime of the melt pool. Therefore, even when Al_2_O_3_ powder is supplied, the particles may not be sufficiently melted or stably captured by the melt pool [39,40]. Consequently, the non-monotonic bead-height behavior observed in this study is interpreted as the combined result of competing effects among heat input, melt pool dynamics, powder capture efficiency, and laser–powder interaction time. However, because direct experimental evidence such as high-speed imaging, wettability measurements, or surface profilometry was not obtained, these mechanisms should be regarded as plausible interpretations rather than conclusively verified causes.

In this study, the deposition behavior of Al_2_O_3_ single beads on a Ti64 substrate and the change in dilution ratio according to energy density were analyzed to derive suitable process conditions for Ti64/Al_2_O_3_ dissimilar deposition.

**Figure 3:**
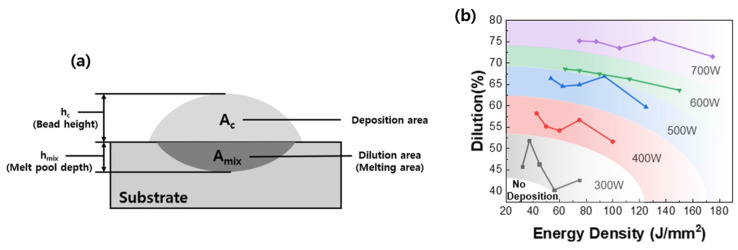
(**a**) Dilution rate schematic and (**b**) Dilution rate according to energy density.

Figure 3a is a schematic diagram illustrating dilution, which occurs as the base material and deposited material melt and mix. The dilution ratio is related to bonding with the substrate because it quantifies the relative contribution of the subsurface melted region to the total bead cross-section. If the dilution ratio is too low, insufficient bonding at the interface between the deposited material and the substrate can lead to dedeposition. Conversely, if the dilution ratio is too high, the deposited bead height decreases and deposition efficiency is reduced. Therefore, a dilution range of 10–30% was adopted in this study as a literature-based reference target for balancing interfacial bonding and deposition efficiency, rather than as a universal optimum value for all material systems [41]. The dilution ratio can be calculated using Equation (2).

(2) $$d=\frac{A_{mix}}{A_{mix}+A_{c}}\times 100\%\approx \frac{h_{mix}}{h_{mix}+h_{c}}\times 100\%$$

where *d* is the dilution ratio, *A_mix_* is the subsurface dilution area, *A_c_* is the superficial deposition area, *h_mix_* is the subsurface dilution height (melt pool depth), and *h_c_* is the superficial deposition height (bead height) [41,42].

Figure 3b shows the relationship between energy density and dilution ratio under the process conditions investigated in this study. The dilution ratio ranged from approximately 40% to 80%; however, the data showed considerable scatter when plotted only as a function of energy density. In particular, similar energy density values produced different dilution ratios depending on laser power. For example, at approximately 75 J/mm^2^, the dilution ratio varied from 42.58% at 300 W–300 mm/min to 64.85% at 500 W–500 mm/min and 75.11% at 700 W–700 mm/min. This indicates that energy density alone was insufficient to fully describe the dilution behavior in the Ti64/Al_2_O_3_ deposition system. Therefore, the dilution results were further interpreted according to laser power. In the 300 W, the dilution ratio was relatively low, ranging from 40.27% to 51.84%, whereas the higher laser power conditions showed increased dilution due to deeper melt pool formation and greater substrate melting. Thus, laser power had a more dominant influence on dilution behavior, while scan speed modified the dilution ratio within each laser power condition. Based on laser cladding and DED-LB literature, a dilution range of 10–30% was used as a reference target for balancing interfacial bonding and deposition efficiency, rather than as a universal optimum value. Although all measured dilution ratios in this study were higher than this reference range, deposition was not achieved at 200 W because the Ti64 substrate was not sufficiently melted. Therefore, the 300 W and 400 mm/min condition, which showed the lowest practical dilution ratio of 40.27% while maintaining successful deposition, was selected as the most suitable condition within the investigated process window.

**Figure 4:**
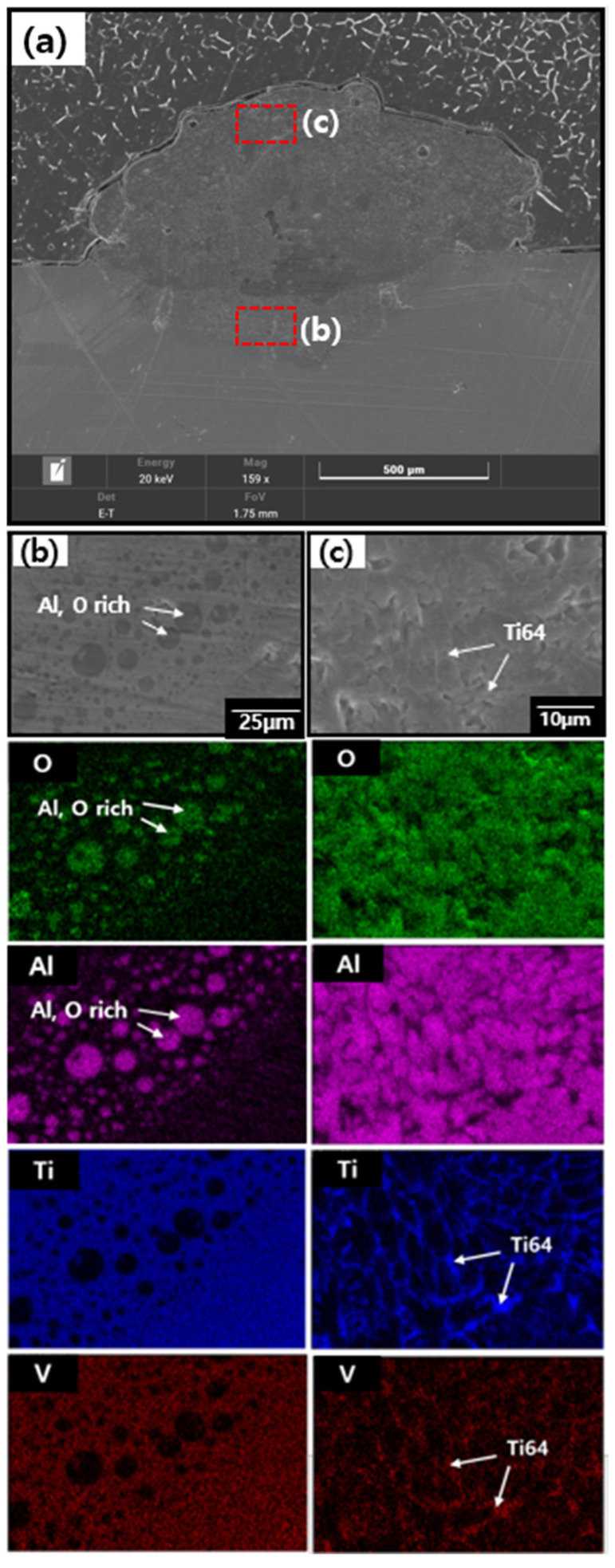
Cross-section of Al_2_O_3_ Bead analyzed using SEM-EDS, Laser power 300 W, Scan speed 400 mm/min, Powder feed rate 3 g/min. (**a**) overall cross-sectional image of the single bead (**b**) cross-sectional image of the melting area (**c**) cross-sectional image of the deposition area.

Figure 4a shows the SEM–EDS-based compositional analysis of a cross-section of a deposited Al_2_O_3_ bead fabricated at a laser power of 300 W and a scan speed of 400 mm/min, which was selected as the most suitable condition based on the dilution analysis in this study. Figure 4b shows a microstructure in which spherical Al_2_O_3_ particles smaller than 15 µm are distributed within the Ti64 melt pool in a single-bead melting region. Based on this, it is determined that most of the Al_2_O_3_ within the Ti64 melt pool has melted. Figure 4c shows that Ti64 was continuously distributed along the Al_2_O_3_ grain boundaries in the deposition zone.

**Figure 5:**
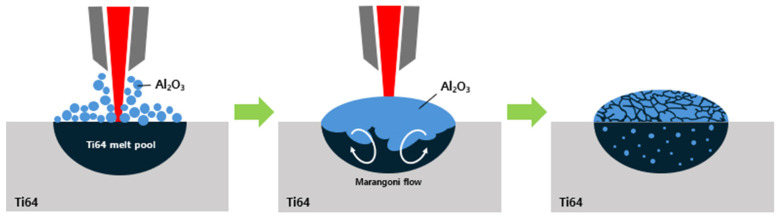
Single Bead deposition schematic.

The formation process of the cross-sectional microstructure of the single bead observed in Figure 4 is schematically depicted in Figure 5.

When a laser is irradiated onto a Ti64 substrate and Al_2_O_3_ powder is supplied, the irradiated Ti64 substrate melts, forming a Ti64 melt pool. Some of the supplied Al_2_O_3_ powder is believed to be incorporated into the melt pool due to strong Marangoni convection and recoil pressure, resulting in dispersion into fine particles. Furthermore, due to the relatively low viscosity and high fluidity of molten Ti64, Al- and O-rich particles presumed to be Al_2_O_3_ may become trapped within the molten metal during solidification, forming a ceramic-rich dispersed phase with particle sizes of less than 15 μm.

Most of the supplied Al_2_O_3_ powder is deposited on the substrate in a molten or partially molten state. During this process, ceramic particles can contact each other and agglomerate, restricting the flow path of the molten metal. According to the literature, Ti64 has a melting point of 1600–1660 °C and solidifies later than Al_2_O_3_, which has a melting point of 2050 °C. Therefore, after Al_2_O_3_-rich regions solidify first, the remaining liquid Ti64 may selectively infiltrate along micropores or capillary passages between the Al_2_O_3_ particles. As a result, fine spherical Al- and O-rich particles smaller than 15 μm were observed within the Ti64 melt pool in the single-bead melting zone, while Ti64 was continuously distributed along the Al_2_O_3_ grain boundaries in the single-bead deposition zone.

**Figure 6:**
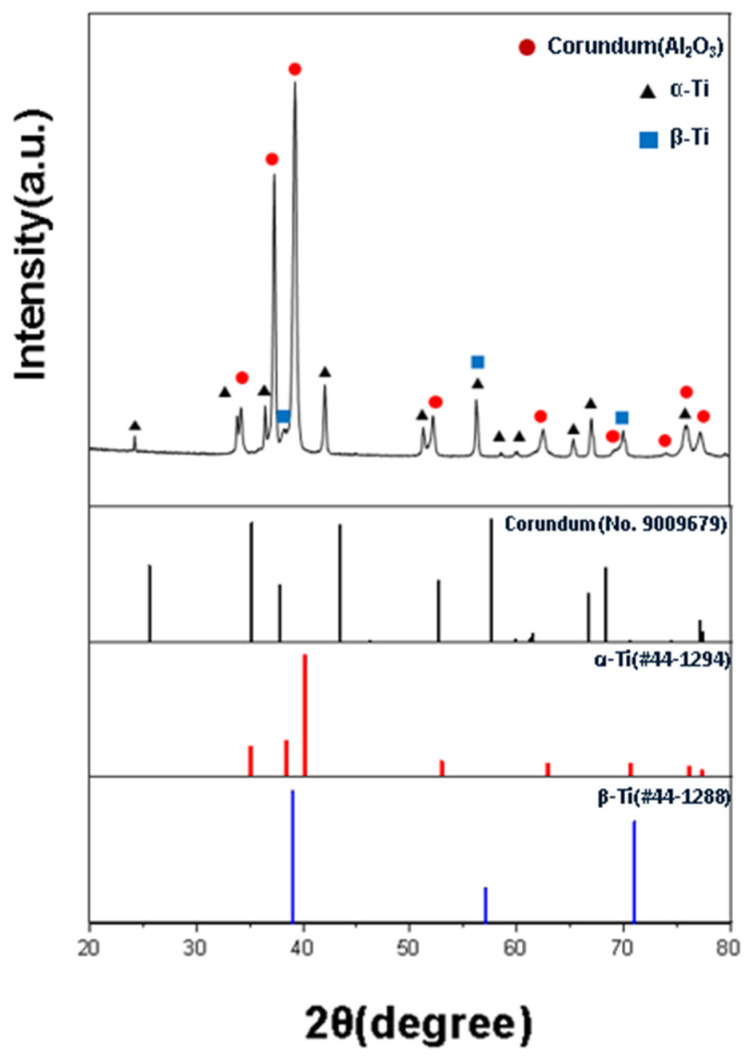
XRD analysis results of Single bead test—laser power: 300 W, scan speed: 400 mm/min.

Even if trace amounts of Ti–Al–O reaction phases are formed during Ti64/Al_2_O_3_ deposition, their possible influence on crack initiation cannot be completely excluded because local differences in thermal expansion coefficient or the formation of brittle phases may promote stress concentration during rapid cooling and solidification. In particular, Al_2_TiO_5_ is known to exhibit large anisotropy in thermal expansion and limited thermal stability, which can lead to internal stress accumulation and microcrack formation during cooling [43]. Therefore, the possible formation of secondary phases such as TiO_2_ or Al_2_TiO_5_ was examined by XRD for the specimen fabricated under the selected condition, as shown in Figure 6. The XRD pattern mainly showed peaks corresponding to Ti64 (α/β-Ti) and corundum-Al_2_O_3_, while no distinct secondary-phase peaks corresponding to TiO_2_ or Al_2_TiO_5_ were observed under the selected condition. However, weak peaks originating from trace reaction phases may have overlapped with the major Ti64 or Al_2_O_3_ peaks or remained below the detection limit of the instrument. Therefore, the present XRD result indicates that no major crystalline TiO_2_ or Al_2_TiO_5_ secondary phases were detected under the selected condition, but it should not be interpreted as completely excluding the possible presence of trace or poorly crystallized reaction phases.

## 4. Conclusions

In this study, a single Al_2_O_3_ bead was deposited on a Ti-6Al-4V (Ti64) substrate using the laser-directed energy deposition (DED-LB) process, and the effects of laser power and scan speed on deposition behavior, bonding characteristics, and microstructural evolution were systematically investigated. The main findings are summarized as follows.

(1) Melt pool depth and bead height were first examined as basic geometric responses to laser power and scan speed. Melt pool depth generally increased with increasing laser power and decreasing scan speed, which was consistent with the increase in areal energy density and indicated enhanced substrate melting. However, these individual geometric trends were not used alone as the criterion for process optimization.
(2) Cracks, pores, and locally detached regions were observed in the deposited beads. The detached regions were attributed to partial material removal during specimen preparation along cracks that had already formed during DED-LB processing. Bead height did not exhibit a monotonic relationship with energy density. Bead formation may have been affected by several coupled mechanisms, including recoil pressure-induced melt pool disturbance, powder scattering, vigorous melt flow under high-power conditions, and insufficient powder melting caused by the shortened melt pool lifetime at high scan speeds.
(3) Therefore, this study proposed a dilution-based process optimization approach rather than relying solely on energy density for Ti64/Al_2_O_3_ dissimilar deposition. Within the explored process window, the condition of 300 W laser power and 400 mm/min scan speed, with the specified powder feed rate, produced a dilution ratio of approximately 40.2%, which was the closest to the target dilution range while still ensuring stable melting of the Ti64 substrate.
(4) Distinct microstructural features were observed in both the fusion and deposition regions under the selected condition. SEM–EDS analysis showed that (i) fine spherical Al- and O-rich particles smaller than 15 μm, presumed to be Al_2_O_3_, were dispersed within the Ti64 melt pool in the fusion zone, and (ii) Ti64 was continuously distributed along the Al_2_O_3_ grain boundaries in the deposition zone. These observations suggest particle entrapment during solidification, followed by metallic infiltration through interparticle channels.
(5) XRD analysis showed dominant α/β-Ti and corundum-Al_2_O_3_ peaks, while no distinct TiO_2_ or Al_2_TiO_5_ secondary-phase peaks were detected under the selected condition. However, the possible presence of trace or poorly crystallized reaction phases cannot be completely excluded because weak diffraction peaks may have overlapped with the major peaks or remained below the detection limit of the instrument.

Overall, this study provides fundamental process conditions and mechanistic insights for the deposition of a single Al_2_O_3_ bead on Ti64 using DED-LB. These findings can serve as a basis for the future design of more stable ceramic-containing tracks, as well as graded or protective ceramic layers on titanium alloys.

## Figures and Tables

**Table 1:** Single-bead deposition results according to DED-LB process variables.

Laser Power (W)	Scan Speed (mm/min)	Energy Density (J/mm^2^)	Melt Pool Depth (μm)	Bead Height (μm)	Dilution (%)
300	300	75	434.58	585.93	42.58
300	400	56.25	345.74	512.72	40.27
300	500	45	318.13	368.55	46.33
300	600	37.5	355.47	330.21	51.84
300	700	32.14	308.71	366.18	45.74
400	300	100	541.58	509.04	51.55
400	400	75	596.64	457.41	56.6
400	500	60	454.98	385.37	54.14
400	600	50	474.2	386.58	55.09
400	700	42.86	477.85	343.35	58.19
500	300	125	773.13	523.46	59.63
500	400	93.75	788.72	391.4	66.83
500	500	75	715.49	387.8	64.85
500	600	62.5	693.89	381.94	64.5
500	700	53.57	653.07	331.41	66.33
600	300	150	986.97	565.47	63.58
600	400	112.5	983.22	501.85	66.21
600	500	90	895.58	433.54	67.38
600	600	75	845.15	393.98	68.21
600	700	64.29	787.52	361.43	68.54
700	300	175	1306.24	522.32	71.44
700	400	131.25	1244.9	402.23	75.58
700	500	105	1101.13	398.57	73.42
700	600	87.5	1061.23	354.19	74.98
700	700	75	985.65	326.67	75.11

## Data Availability

The original contributions presented in the study are included in the article; further inquiries can be directed to the corresponding author.

## Nomenclature

DED-LB, laser-directed energy deposition; Ti64, Ti-6Al-4V alloy; Al_2_O_3_, aluminum oxide/alumina; FGM, functionally graded material; OM, optical microscopy; SEM, scanning electron microscopy; EDS, energy dispersive X-ray spectroscopy; XRD, X-ray diffraction; *P*, laser power; *V*, scan speed; *f*, laser beam diameter; *E*, areal energy density; *d*, dilution ratio; *A_mix_*, subsurface dilution area; *A_c_*, superficial deposition area; *h_mix_*, subsurface dilution height/melt pool depth; *h_c_*, superficial deposition height/bead height; 2θ, diffraction angle; CTE, coefficient of thermal expansion.
